# Quadriceps and Respiratory Muscle Fatigue Following High-Intensity Cycling in COPD Patients

**DOI:** 10.1371/journal.pone.0083432

**Published:** 2013-12-06

**Authors:** Damien Bachasson, Bernard Wuyam, Jean-Louis Pepin, Renaud Tamisier, Patrick Levy, Samuel Verges

**Affiliations:** 1 Grenoble Alpes University, HP2 Laboratory, Grenoble, France; 2 INSERM, U1042, Grenoble, France; 3 CHU, Grenoble Locomotor Unit, Reeducation & Physiology, Clinical Physiology, Sleep and Exercise, Grenoble, France; Clinica Universidad de Navarra, Spain

## Abstract

Exercise intolerance in COPD seems to combine abnormal ventilatory mechanics, impaired O_2_ transport and skeletal muscle dysfunction. However their relatie contribution and their influence on symptoms reported by patients remain to be clarified. In order to clarify the complex interaction between ventilatory and neuromuscular exercise limiting factors and symptoms, we evaluated respiratory muscles and quadriceps contractile fatigue, dynamic hyperinflation and symptoms induced by exhaustive high-intensity cycling in COPD patients. Fifteen gold II-III COPD patients (age = 67±6 yr; BMI = 26.6±4.2 kg.m^-2^) performed constant-load cycling test at 80% of their peak workload until exhaustion (9.3±2.4 min). Before exercise and at exhaustion, potentiated twitch quadriceps strength (Q_tw_), transdiaphragmatic (P_di,tw_) and gastric (P_ga,tw_) pressures were evoked by femoral nerve, cervical and thoracic magnetic stimulation, respectively. Changes in operational lung volumes during exercise were assessed *via* repetitive inspiratory capacity (IC) measurements. Dyspnoea and leg discomfort were measured on visual analog scale. At exhaustion, Q_tw_ (-33±15%, >15% reduction observed in all patients but two) and P_di,tw_ (-20±15%, >15% reduction in 6 patients) were significantly reduced (P<0.05) but not P_ga,tw_ (-6±10%, >15% reduction in 3 patients). Percentage reduction in Q_tw_ correlated with the percentage reduction in P_di,tw_ (r=0.66; P<0.05). Percentage reductions in P_di,tw_ and P_ga,tw_ negatively correlated with the reduction in IC at exhaustion (r=-0.56 and r=-0.62, respectively; P<0.05). Neither dyspnea nor leg discomfort correlated with the amount of muscle fatigue. In conclusion, high-intensity exercise induces quadriceps, diaphragm and less frequently abdominal contractile fatigue in this group of COPD patients. In addition, the rise in end-expiratory lung volume and diaphragm flattening associated with dynamic hyperinflation in COPD might limit the development of abdominal and diaphragm muscle fatigue. This study underlines that both respiratory and quadriceps fatigue should be considered to understand the complex interplay of factors leading to exercise intolerance in COPD patients.

## Introduction

Dyspnea but also leg discomfort are described by patients with chronic obstructive pulmonary disease (COPD) as the primary symptoms responsible for exercise limitation [1–3]. Pathophysiologic mechanisms involved in this limitation seem to combine abnormal ventilatory mechanics, impaired oxygen (O_2_) transport and skeletal muscle dysfunction [2,4]. Their relative contribution to exercise intolerance remains extensively debated [5]. 

In peripheral locomotor muscle, intrinsic muscle alterations (e.g. fiber type shift toward type II/IIX, reduced oxidative enzyme activities) contribute to higher exercise-induced contractile fatigue in patients with COPD [2,6]. Enhanced quadriceps fatigability appeared to be present in patients even with preserved strength [6] and may occur in all [3,7] or only a fraction [2] of patients. Enhanced leg discomfort during exercise may be related to impaired quadriceps contractility [2,3]. Butcher et al. [8] recently showed that patients with larger level of hyperinflation exhibit less quadriceps fatigue suggesting a relationship between respiratory muscle work and locomotor muscle fatigue [3]. 

In healthy subjects, inspiratory and expiratory muscle contractile fatigue develops during exercise [9,10] and can limit performance [11,12]. In COPD, increased work of breathing results in diaphragm remodeling toward a more fatigue-resistant phenotype and hyperinflation induces structural adaptation to cope with unfavorable strength-length relationship [13]. Despite these adaptations, diaphragm from COPD patients exhibits impaired contractility [14] and is more likely to develop injuries with exertion [15]. However, objective diaphragm fatigue has not been consistently reported following exhaustive exercise, although some patients may exhibit significant amount of fatigue [16–18]. Concerning expiratory muscles, it is not clear whether weakness is present [19] or absent [20] in COPD patients . Only one study [17] investigated expiratory muscle fatigue following maximal exercise and reported a significant reduction in abdominal muscle contractility. While dyspnea and mechanical ventilatory constraint seem to be related [4], no data exist regarding the relationship between respiratory muscle fatigue, dynamic hyperinflation and dyspnea during exercise.

 Hence, the presence of locomotor and respiratory muscle fatigue during exhaustive exercise in COPD patients and their relationship with dynamic hyperinflation and symptoms remain be further clarified. Accordingly, we evaluated quadriceps, diaphragm and abdominal muscle fatigue using non-volitional strength assessments *via* magnetic neurostimulation [21,22], dynamic hyperinflation and symptoms induced by exhaustive high intensity constant-workload cycling. We hypothesized that i) the amount of dynamic hyperinflation during exercise would impact on respiratory muscle fatigue, and ii) patients with significant amount of muscle fatigue would report the greatest level of dyspnea (patients with respiratory muscle fatigue) or leg discomfort (patients with quadriceps fatigue).

## Methods

### Ethics statement

This study was conducted according to the Declaration of Helsinki with approval from the local Ethics Committee (Comité de protection des personnes Sud-Est V) and all patients gave informed written consent.

### Patients

Fifteen patients with GOLD II-III COPD (twelve men and three women) were included in this study. According to the revised GOLD classification [23], seven patients were GOLD group C, six patients were GOLD group B and two patients were GOLD group A. All subjects underwent a rigorous characterization process including medical history, physical examination, and ECG at rest and during a standard incremental cardiopulmonary cycling test. Patients with cardiovascular, musculoskeletal, neurological diseases or any other condition that could alter their capacity to perform the exercise test were not included in the study. None of the patients were on long-term oxygen therapy and none had severe exacerbation during the 6 months before the study. All patients had stopped smoking were taking bronchodilators and did not modify their treatments during the study. All participants were sedentary, defined as no regular physical activity or enrollment in a rehabilitation program for at least the prior 6 months. 

### Study design

At screening, spirometry, lung volumes and resting arterial blood gases were measured according to standard guidelines [24]. On a first visit, patients performed a maximal incremental exercise test on a computer-controlled electrically braked cycle ergometer (Ergometrics 800, Ergoline, Bitz, Germany) with breath-by-breath gas analysis and electrocardiogram (Medisoft, Dinant, Belgium) to determine peak workload capacity (W_peak_) and O_2_ consumption (V̇O_2,peak_). Initial workload was 20 W followed by 2-min increments of 10 W until exhaustion. A fingertip blood sample was obtained 3 min after exhaustion and analyzed for lactate concentration (NOVA+, Nova Biomedical Corporation, Waltham MA, USA). On a second visit (at least 48 h after the first visit and at the latest one week after), subjects performed a constant-load cycling test at 80% of W_peak_ until exhaustion after a 2-min warm-up at 20 W. Dyspnoea and leg discomfort were assessed each min with a standard 100 mm visual analog scale and primary symptom for stopping exercise was reported. Voluntary inspiratory capacity (IC), end-expiratory (EELV) and end-inspiratory lung volume (EILV), minute ventilation (V̇E), tidal volume (V_T_), inspiratory (IRV) and expiratory (ERV) reserve volume were measured at rest before cycling and each minute during exercise in order to assess dynamic hyperinflation [4]. At each time point, patients performed two IC manoeuvers and the highest value was used for analysis. Before the test (Baseline) and immediately after exhaustion, non-volitional quadriceps and respiratory muscles strength assessments were performed (see below). Data from the constant-load cycling test are presented as 30 second averages or the nearest IC measurement corresponding to Baseline, 5 min after the start of exercise and immediately before exhaustion. 

### Quadriceps and respiratory neuromuscular function

#### Quadriceps

Voluntary strength and mechanical evoked responses to femoral nerve magnetic stimulation (FNMS) was measured as described elsewhere [21,25]. Single (twitch) stimulations were performed at maximum stimulator output. Optimal stimulation site allowing maximal quadriceps twitch (Q_tw_) strength was determined and marked on the skin. Patients performed three 5-s maximal voluntary contractions (MVC) with 30 s of rest between each MVC allowing full muscle potentiation [26] prior to a series of six stimulations. After three stimulations, another 5-s MVC was performed to prevent depotentiation. The average amplitude (baseline to peak) of all twitches was calculated. Immediately after exhaustion, the same procedure was repeated. The procedure for Q_tw_ measurement before and after exercise took 4 to 5 min.

#### Respiratory muscles

Cervical and thoracic magnetic stimulations were performed using a circular 90-mm coil powered by a Magstim 200 stimulator (MagStim) as described in a previous work [22]. Oesophageal (P_oes_) and gastric (P_ga_) pressures were measured by 10-cm balloon catheters, connected separately to differential pressure transducers (model DP45-30; Validyne, Northridge, CA). Transdiaphragmatic pressure (P_di_) was obtained by online subtraction of P_oes_ from P_ga_. Cervical magnetic stimulation of the phrenic nerves was performed while subjects were seated comfortably in a chair with the center of the coil positioned at the seventh cervical vertebra [27]. Thoracic stimulation of the nerve roots innervating abdominal muscles was performed with the center of the coil positioned at the intervertebral level T10 [22]. The best spot allowing the maximal twitch pressures (P_di,tw_ during cervical stimulation and P_ga,tw_ during thoracic stimulation) was determined with minor adjustments and then marked on the skin. The order of cervical and thoracic stimulations was randomized between subjects but was the same at before and after exercise for a given subject. To avoid the confounding effect of potentiation [28,29], subjects performed three 5-s maximal inspiratory efforts from functional residual capacity (FRC, for cervical stimulation) or three 5-s maximal expiratory efforts from total lung capacity (TLC, for thoracic stimulation) against a closed airway prior to a series of six stimulations. After three stimulations, another 5-s maximal voluntary contraction was performed. All stimuli were delivered at FRC, with the airway occluded. To ensure the same lung volume at all times before and after exercise, the experimenter checked that for each subject, pre-stimulation P_oes_ ranged at the same level immediately before each stimulation [30]. Recordings that showed changes in pre-stimulation P_oes_ were rejected post hoc. For data analysis, the average amplitude (baseline to peak) of all remaining twitches (at each stimulation site) was calculated. P_oes,tw_ /P_ga,tw_ ratio during cervical stimulation was calculated as an index of extra-diaphragmatic inspiratory muscle fatigue [27]. Four patients were not considered for analysis because of i) inability to tolerate balloon catheters (n=2), ii) methodological issues regarding balloons catheters (technical problem or inability to consistently stimulate at FRC, n=2). The procedure for P_di,tw_ and P_ga,tw_ measurements took 5 to 6 min. It was performed immediately after Q_tw_ measurement to allow some reduction in ventilation and dyspnoea after exhaustion. Strength and pressure signals were digitized (Powerlab, ADInstruments, Sydney, Australia) and recorded simultaneously on a computer (Labchart; ADInstruments; sampling frequency: 2 kHz). A >15% fall in evoked muscular responses (P_di,tw_, P_ga,tw_ or Q_tw_) was used as a threshold indicating significant amount of fatigue [2].

### Statistical Analysis

All descriptive statistics presented are mean values ± SD. Normal distribution and homogeneity of variances analysis were confirmed using the Kolmogorov-Smirnov and Skewness test, respectively. Paired t-tests were conducted to compare neuromuscular parameters before and after exercise. To compare changes in cardiorespiratory variables during the constant-load cycling test (at Baseline, 5min after the beginning of the test and at exhaustion), we used one-way repeated measures ANOVA and t-tests with Tukey’s test for post-hoc analysis. Pearson’s correlations were used to determine relationship between variables. Statistical analysis was performed with a statistical software package (NCSS, Kaysville, Utah USA). The alpha level was set at 0.05 for all tests.

## Results

### Patient characteristics

The main characteristic of the subjects are summarized in Table 1. BMI ranged between 20.5 and 35.6 kg.m^-2^ with seven patients having normal weight, five overweight patients and three obese patients. Patients had moderate to severe airflow obstruction (forced expiratory volume in one second (FEV_1_) ranged between 40 and 71% of predicted values and FEV_1_/forced vital capacity (FVC) ranged between 37 and 68 %), a significant level of thoracic distension (residual volume (RV)/total lung capacity (TLC) ranged between 38 and 67 %). Data from the incremental cycling test are shown in Table 2. At peak exercise, the expiratory exchange ratio >1, the absence of ventilatory reserve (V̇E/maximal voluntary ventilation in 12s (MVV) >100%) and the high symptoms scores confirmed maximal exercise testing [31].

**Table 1:** Patients’ characteristics.

**Variables**	**Absolute**	**% Predicted**
**Age (years)**	67 ± 6	/
**BMI (kg.m^-2^)**	26.6 ± 4.2	/
**FVC (l)**	3.04 ± 0.78	84 ± 14
**FEV_1_ (l)**	1.54 ± 0.42	54 ± 11
**FEV_1_/FVC (%)**	51± 9	68 ± 13
**MVV (l.min^-1^)**	58 ± 19	59 ± 16
**IC (l)**	2.6 ± 0.4	91 ± 13
**IC/TLC (%)**	35 ± 6	/
**RV (l)**	4.6 ± 0.7	192 ± 40
**TLC (l)**	7.75 ± 1.0	122 ± 18
**RV/TLC (%)**	60 ± 5	143 ± 22
**PO_2_ (mmHg)**	71 ± 12	/
**PCO_2_ (mmHg)**	35 ± 5	/
**pH**	7.43 ± 0.05	/
**SpO_2_ (%)**	94.0 ± 3.3	/
**MIP (cmH_2_O)**	93 ± 22	101 ± 28
**SNIP (cmH_2_O)**	69 ± 20	75 ± 22
**MEP (cmH_2_O)**	132 ± 40	80 ± 34
**MVC (kg)**	50 ± 13	88 ± 23

Values are mean ± SD. BMI, body mass index; FVC, forced vital capacity; FEV_1_, forced expiratory volume in 1 s; MVV, maximal voluntary ventilation measured in 12 s; IC, inspiratory capacity; RV, residual volume; TLC, total lung capacity; PO_2_, partial arterial pressure in O_2_; PCO_2_, partial arterial pressure in CO_2_; SpO_2_, arterial O_2_ saturation by pulse oximetry; MIP, maximal inspiratory pressure; MEP, maximal expiratory pressure; SNIP, sniff nasal inspiratory pressure; MVC, maximal voluntary quadriceps strength.

**Table 2:** Peak physiologic response during the maximal incremental cycling test.

**Variables**	**Absolute**	**% Predicted**
**W_peak_ (W)**	92 ± 17	58 ± 11
**O_2,peak_ (ml.min^-1^.kg^-1^**)	19.5 ± 2.5	70 ± 12
**V̇O_2,peak_ (l.min^-1^**)	1.4 +0.3	70 ± 14
**V̇CO_2_ (l.min^-1^)**	1.6 ± 0.3	/
**V̇E (l.min^-1^)**	64 ± 17	/
**V̇E/ O_2_**	44 ± 11	/
**V̇E / CO_2_**	40 ± 6	/
**V̇E /MVV (%)**	110 ± 22	/
**RER**	1.1 ± 0.1	/
**SpO_2_ (%)**	94 ± 2.9	/
**HR_max_, (beat.min^-1^)**	145 ± 28	94 ± 17
**[La]_max_ (mmol.l^-1^)**	4.9 ± 1.1	/
**Limb discomfort VAS score**	8.2 ± 1.6	/
**Dyspnea discomfort VAS score**	8.5 ± 1.8	/
**Reason for stopping**		
Dyspnea	7	/
Limb discomfort	5	/
Both	3	/

Values are mean ± SD; W_peak_, peak workload; **V̇**O_2,peak_, peak oxygen consumption; **V̇**E, minute ventilation; **V̇**CO_2_, carbon dioxide production; MVV, maximal voluntary ventilation measured in 12 s; SpO_2_, arterial O_2_ saturation by pulse oximetry; RER, respiratory exchange ratio; HR _max_, maximal heart rate; [La]_max_, maximal blood lactate concentration; VAS, visual analog scale.

### Physiologic responses to exhaustive constant-load cycling

**Figure 1:**
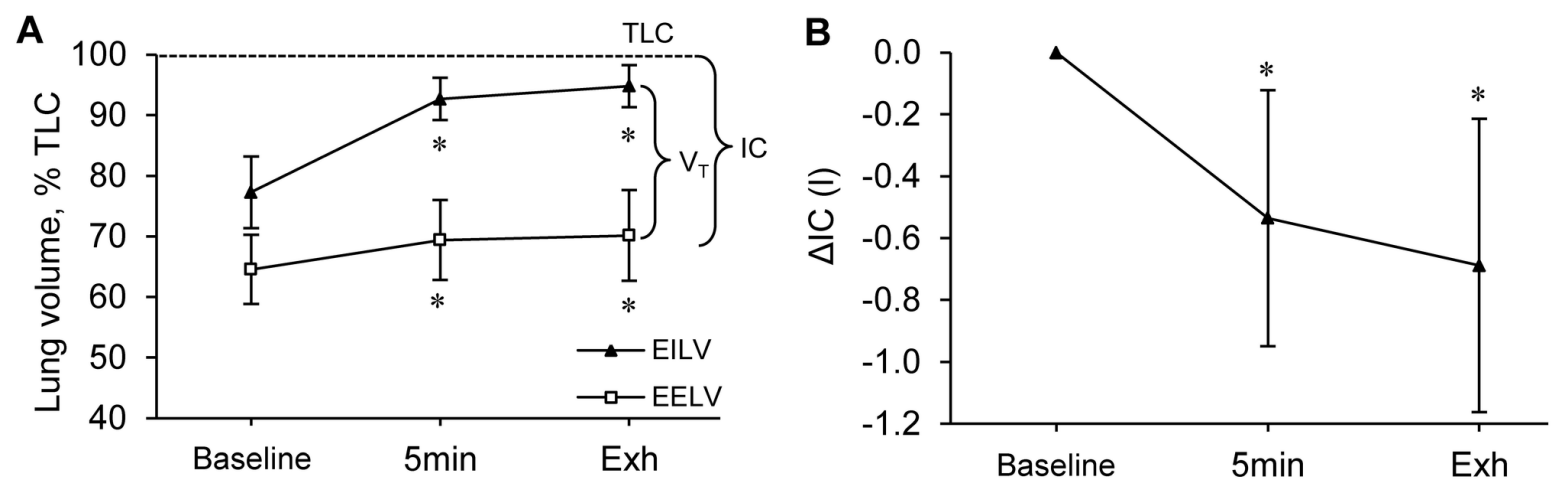
End-expiratory (EELV) and end-inspiratory (EILV) lung volumes as a percentage of total lung capacity (TLC), tidal volume (V_T_) and inspiratory capacity (IC) before (Baseline), 5 minutes after the start of constant-load cycling (5 min) and at exhaustion (Exh) (Panel A). Changes in IC during the same conditions (Panel B). *, significant differences from Baseline (P<0.05).

Mean exercise duration was 9.3±2.4 min and the intensity was 95±21 W. Physiological responses to the final 30 s of exercise and exercise-induced symptoms are shown in Table 3. V̇O_2_, heart rate (HR), and **V̇**E progressively increased to reach similar levels than at the end of the maximal incremental cycling test. At Exhaustion, mean reduction in SpO_2_ was -5±3 % (range between -1 and -11%) and no patient had SpO_2_ below 85%. The primary symptom for stopping exercise was not uniform and was similar to those reported during the maximal incremental cycling test in all patients but three. Changes in operational lung volumes during exercise are shown in Figure 1. From rest to peak exercise, IC decreased by 19 ±12% of predicted value and IRV was critically reduced (<1 l) in all patients but one. 

**Table 3:** Physiologic response at exhaustion during the constant-load cycling test.

**Variables**	**Absolute**	**% of Max**
**V̇O_2_ (l.min^-1^)**	1.3 ± 0.3	96 ± 18
**V̇CO_2_ (l.min^-1^)**	1.4 ± 0.4	94 ± 14
**V̇E (l.min^-1^**)	60 ± 17	94 ± 12
**V̇E /O_2_**	41 ± 6	104 ± 14
**V̇E /CO_2_**	42 ± 5	106 ± 9
**V̇E /MVV (%)**	107 ± 23	98 ± 12
**RER**	1.1 ± 0.1	98 ± 7
**VT (l)**	1.57 ± 0.43	104± 16
**TI/T_tot_**	0.44 ± 0.15	99 ± 4
**f_R_ (breath.min-^1^)**	35 ± 4	97 ± 8
**SpO_2_ (%)**	94 ± 4	99 ± 2
**HR (beat.min^-1^)**	133 ± 24	96 ± 7
**Dyspnea discomfort VAS score**	8.6±1.7	106 ± 29
**Limb discomfort VAS score**	8.0±2.1	98 ± 22
**Reason for stopping**		
Dyspnea	4	/
Limb discomfort	5	/
Both	5	/

Values are mean ± SD. VT, tidal volume; TI, inspiration time; T_tot_, total respiratory cycle time; F_R_, respiratory frequency; % Max, values expressed as percentage of peak values during the maximal incremental cycling test. See table 2 for other abbreviations

### Neuromuscular fatigue induced by exhaustive constant-load cycling

#### Quadriceps

**Figure 2:**
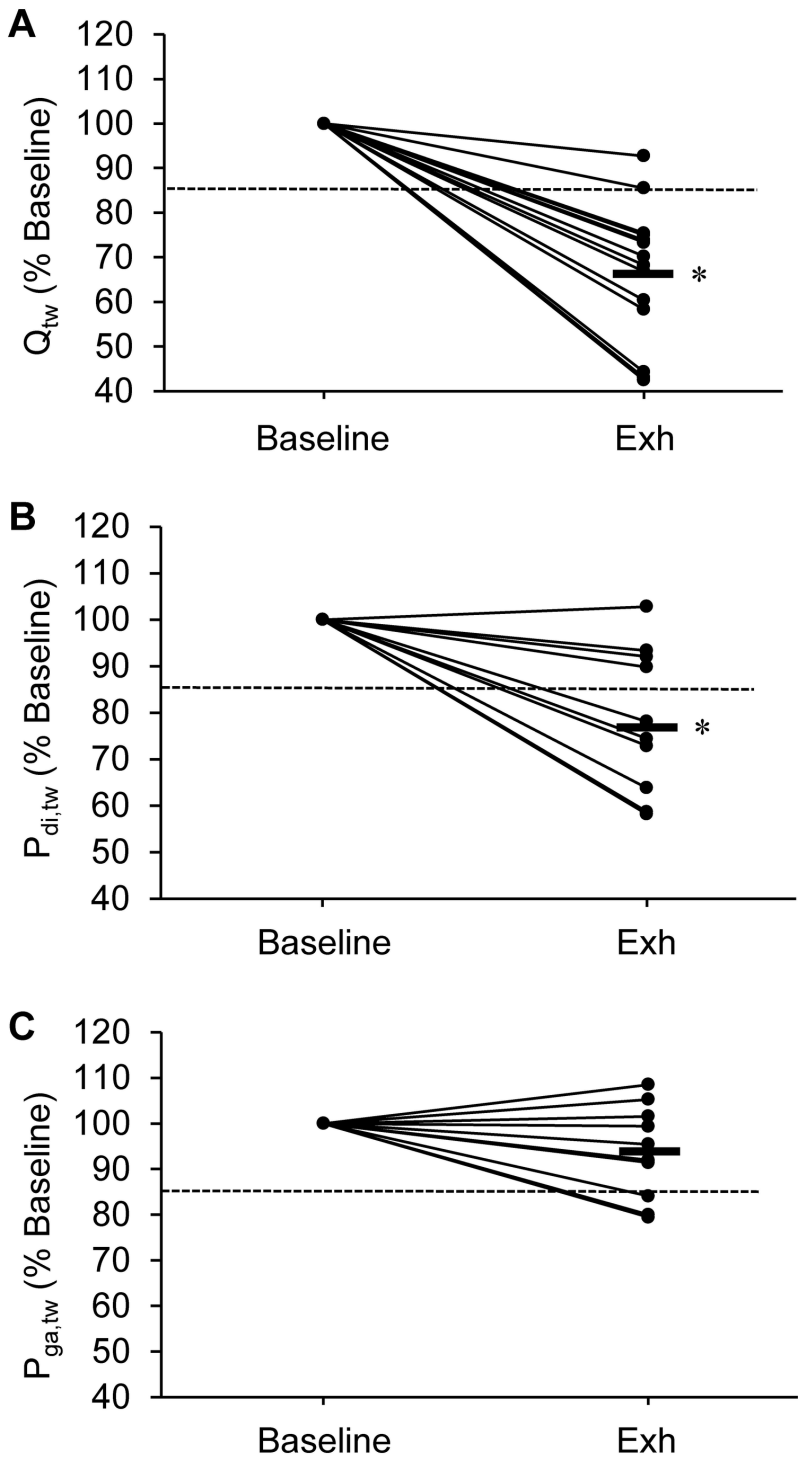
Individual changes in quadriceps strength evoked by femoral magnetic stimulation (Q_tw_; Panel A), transdiaphragmatic pressure evoked by cervical magnetic stimulation (P_di,tw_; Panel B) and gastric pressure evoked by thoracic magnetic stimulation (P_ga,tw_; Panel C) from before (Baseline) the constant-load cycling to immediately after exhaustion (Exh). The thick line indicates the group average reduction. The dotted line indicates the 15% reduction threshold for significant amount of muscle fatigue. *, significant group difference from Baseline (P<0.05).

Q_tw_ at Baseline and after exhaustion is shown in Table 4 and in Figure 2. Immediately after Exhaustion, Q_tw_ was significantly reduced (-34±15%) and all patients but two had a reduction >15%. The percentage reduction in Q_tw_ did not correlate with the reduction in IC at exhaustion (r=0.26; P=0.20), symptoms at exhaustion (dyspnoea: r=0.07; P=0.79; leg discomfort: r=0.15; P=0.59) or exercise duration (r=-0.22; P=0.43).

**Table 4:** Quadriceps and respiratory muscle responses evoked by femoral, cervical and thoracic magnetic stimulation, respectively, before (Baseline) and immediately after exhaustive constant-load cycling.

	**Variables**	**Baseline**	**Exhaustion**	**P value**
**Femoral magnetic neurostimulation** (n=15)	Q_tw_ (kg)	16.2±4.8	11.0 ±4.7	<0.001
**Cervical magnetic neurostimulation** (n=11)	P_di,tw_ (cmH_2_O)	17.6 ± 5.2	14.1 ± 5.1	<0.001
	P_oes_/P_ga_	1.24 ± 0.70	1.10 ± 0.44	0.39
**Thoracic magnetic neurostimulation** (n=11)	P_ga,tw_ (cmH_2_O)	28.5 ± 16.4	26.7 ± 13.7	0.20

Values are mean ± SD; Q_tw_, quadriceps twitch strength; P_di,tw_, diaphragmatic twitch pressure; P_oes_/P_ga_, ratio of the oesophageal and gastric twitch pressures; P_ga,tw_, gastric twitch pressure.

#### Inspiratory muscle

**Figure 3:**
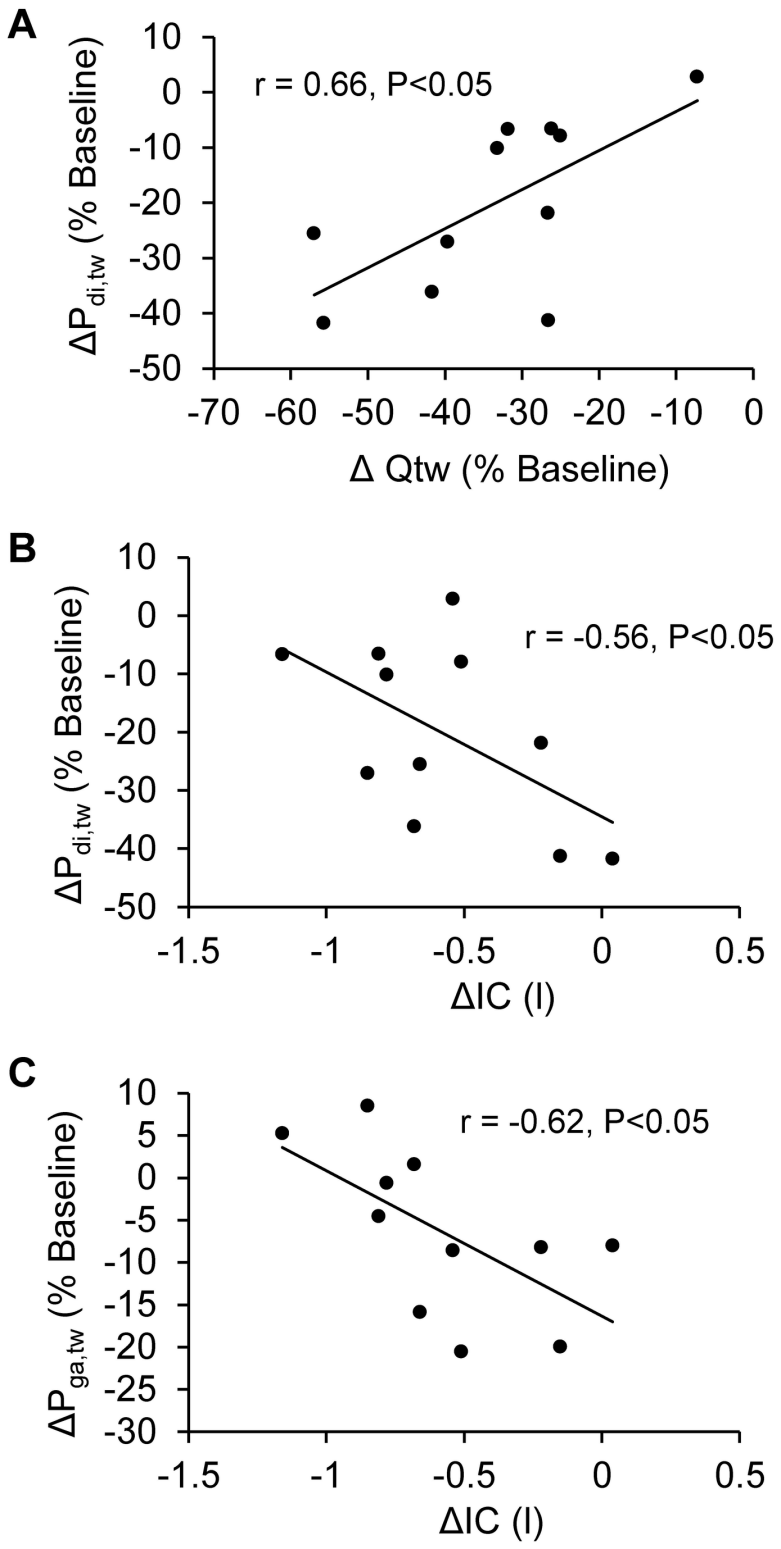
Correlation of the percentage reduction from baseline in transdiaphragmatic pressure evoked during cervical magnetic stimulation (ΔP_di,tw_) with the percentage reduction from Baseline in quadriceps strength evoked during femoral magnetic stimulation immediately after exhaustive constant load cycling (ΔQ_tw_; Panel A). Correlation of ΔP_di,tw_ (Panel B) and the percentage reduction from Baseline of gastric pressure evoked during thoracic magnetic stimulation immediately after exhaustive constant load cycling (ΔP_ga,tw_ ; Panel C) with the reduction in IC at exhaustion (ΔIC).

P_di,tw_ at Baseline and after exhaustion is shown in Table 4 and in Figure 2. P_di,tw_ at Baseline correlated with percentage of predicted TLC (r=-0.67; P<0.05) and RV (r=-0.72; P<0.05). Immediately after exhaustion, P_di,tw_ was significantly reduced (-20±15%) and six patients had a reduction >15%. No significant change in P_oes_/P_ga_ was observed (P=0.71). P_oes_ immediately before stimulations was similar before and after exercise (mean change was 0.04±2.40 cmH_2_O; P=0.95). The percentage reduction in P_di,tw_ was significantly smaller than the percentage reduction in Q_tw_ (P<0.001) and correlates with it (Figure 3A). The percentage reduction in P_di,tw_ correlated with the reduction in IC at 5 min (r=-0.57; P<0.05) and at exhaustion (Figure 3B), but not with symptoms (at exhaustion: dyspnoea r=0.20; P=0.18; leg discomfort r=-0.23; P=0.67) or exercise duration (r=-0.28; P=0.31).

#### Expiratory muscle

P_ga,tw_ at Baseline and after exhaustion is shown in Table 4 and in Figure 2. After exhaustion, the reduction in P_ga,tw_ (-6±10%) was not statistically significant at the group level (P=0.29). Three patients had a percentage reduction >15%. P_oes_ immediately before stimulations was similar at Baseline and after exhaustion (mean change was -0.98±1.69 cmH_2_O; P=0.51)). The percentage reduction in P_ga,tw_ correlated with the reduction in IC at 5 min (r=-0.60; P<0.05) and at exhaustion (Figure 3C) but not with the percentage reduction in P_di,tw_ (r=0.11; P=0.71) and Q_tw_ (r=0.10; P=0.76), symptoms (at exhaustion: dyspnoea r=0.07; P=0.62; leg discomfort r=0.10; P=0.80) or exercise duration (r=-0.13; P=0.57).

No significant difference was found between GOLD II (n=8, GOLD groups A and B) and GOLD III (n=7, GOLD groups C) patients regarding exercise-induced percentage reduction in P_di,tw_, P_ga,tw_ and Q_tw_ (all P>0.35).

## Discussion

This study is the first to evaluate dynamic hyperinflation, symptoms, locomotor and respiratory muscle fatigue induced by exhaustive high intensity constant load cycling within the same group of patients with COPD. The main findings are as follows: i) significant reductions in evoked quadriceps and diaphragm responses were observed in most of the patients while only few patients had reduced abdominal contractility following exercise, ii) the reduction in IC (i.e. an index of dynamic hyperinflation) during exercise negatively correlated with the percentage reduction in P_di,tw_ and P_ga,tw_ immediately after exhaustion and iii) no correlation was observed between the amount of muscle fatigue and symptoms of dyspnoea or leg discomfort.

### Neuromuscular fatigue induced by exhaustive constant-load cycling

#### Quadriceps fatigue

Significant Q_tw_ reduction after exhaustion reveals exercise-induced impairment of quadriceps contractility. At the group level, this reduction in Q_tw_ was consistent with reductions we and others previously observed in healthy subjects (~35-40% versus ~34% in the present work) following similar constant load exhaustive exercise [32,33]. Although we did not measure membrane excitability, we believe that this reduction is mainly related to contractile fatigue since alteration of nerve conduction has never been reported in COPD after exhaustive cycling [3,7]. The occurrence of significant amount of quadriceps fatigue (i.e. defined as a >15% reduction in Q_tw_ [34]) was higher in our, Amman et al. [3], and Mador et al.[7] studies (near 100%) compared to studies of Saey et al. [2,34] and Butcher et al. [8] (~50, ~69%, and ~64%, respectively) which suggested that a significant amount of COPD patients does not develop quadriceps fatigue during cycling. In the study of Amann et al. [3] and Mador et al. [7], the endurance time was similar but the intensity of the constant-load exercise was lower (~50% and ~60% of W_peak_, respectively) compared to the present study. Larger patients BMI (~35 *versus* ~27 kg.m^-2^ in the present study) and arterial SpO_2_ desaturation (at rest ~86% *versus* ~95% in our work) in Amann et al. [3] and greater airflow obstruction and exercise intolerance (FEV_1_ and **V̇**O_2,peak_ ~40% of predicted values) in Mador et al. [7] may partly explain similar prevalence of quadriceps fatigue despite lower exercise intensity compared to the present study. In the studies of Saey et al. [2,34] and Butcher et al. [8], similar constant-load relative exercise intensity but shorter endurance time (~5-6 min *versus* ~11 min in the present study) may be responsible, at least in part, for the lower occurrence of significant quadriceps contractile fatigue. Different quadriceps function assessment procedures (e.g. 10 min delay after exhaustion [2,34] *versus* 5min in the present work; *vastus lateralis* motor point stimulation [8] *versus* supramaximal femoral nerve stimulation in the present study) might also have allowed a significant recovery in some patients or underestimation of muscle fatigue in previous studies. We did not observe a significant correlation between dynamic hyperinflation and quadriceps fatigue in contrast to Butcher et al. [8]. The fact that patients recruited in the later study participated in a rehabilitation program prior to the study whereas patients included in the present study did not is another factor that might explain differences between both studies. All together these results suggest that the diversity of patients’ phenotypes, exercise intensity and duration, and differences in neuromuscular fatigue assessment procedures are important factors influencing the development of quadriceps fatigue during cycling in COPD patients. 

#### Diaphragm fatigue

Significant P_di,tw_ reduction immediately after exhaustion suggests exercise-induced impairment in diaphragm contractility while unchanged P_oes_/P_ga_ values indicates predominant diaphragm fatigue [27]. Similar reduction in P_di,tw_ was previously observed in healthy subjects (~15-20% *versus* ~20% in the present work) following constant load exhaustive exercise [10,35–37]. In COPD, this P_di,tw_ reduction contrasts with previous studies having reported unchanged P_di,tw_ after exhaustive constant-load cycling at ~65 % W_peak_ [18], maximal incremental cycling [17] and walking [16]. In these studies, unpotentiated stimulations were used and the authors made the assumption that 10 [18] or 20 [16,17] minutes of rest would be sufficient to avoid exercise-induced twitch potentiation after exercise. However 6 of the 12 patients enrolled in the study of Mador et al. [18] showed increased evoked diaphragmatic response with cervical magnetic stimulation after a similar exercise which might be explained by exercise-induced twitch potentiation. The later may have also reduced the amplitude of twitch reduction observed after exercise in the other patients. In addition delayed neuromuscular assessment after exercise may allow substantial recovery as recently shown for the quadriceps by Froyd et al. [38]. In the present work, we measured fully potentiated responses both before and within 10 min after exercise by using several maximal manoeuvers immediately before stimulations to limit potential underestimation of contractile fatigue [28,29] and because potentiated twitches have been reported to be more reproducible and more sensitive to muscle fatigue [26]. 

During exercise, patients exhibit heterogeneous levels of hyperinflation (Figure 1) as previously observed in large sample of COPD patients [4]. Interestingly, patients with limited reduction in IC during exercise showed greater diaphragm fatigue than patients with larger reduction in IC (Figure 3B). In patients with large hyperinflation, the diaphragm might make a limited contribution to inspiratory muscle work because of impaired pressure-generating capacity induced by diaphragm flattening [39,40]. As a consequence, diaphragm fatigue might be less likely to develop in these patients and recruitment of extra diaphragmatic muscle during inspiration may be enhanced to cope with increased elastic loading [40]. However, since no significant correlation was found between the IC reduction and the P_oes_/P_ga_ ratio, this hypothesis remained to be confirmed with other indicators of extra diaphragmatic inspiratory muscle recruitment. Shortened length of the diaphragm in patients with large dynamic hyperinflation might also reduce the development of fatigue [41]. Smaller amount of diaphragm fatigue compared to quadriceps fatigue probably illustrates the striking differences between quadriceps and diaphragm phenotypes (e.g. greater aerobic capacities in diaphragm *versus* quadriceps [42]). Major alterations in muscle morphology and bioenergetics in COPD patients may be responsible for the increased quadriceps muscle fatigability in COPD patients [3]. The significant correlation between the percentages reduction in Q_tw_ and P_di,tw_ (Figure 3A) suggests that i) common mechanisms underlie respiratory and locomotor muscle fatigue development in COPD patients (e.g. hypoxemia, low-grade inflammation, oxidative stress) and/or ii) a relationship between respiratory and locomotor muscle fatigue through a metaboreflex as recently suggested [3]. 

#### Abdominal muscle fatigue

P_ga,tw_ after exhaustion was not significantly reduced during thoracic magnetic stimulation at the group level suggesting preserved abdominal muscle contractility following exhaustive cycling in most COPD patients. This result contrast with greater reduction in P_ga,tw_ previously reported in healthy subjects following similar exercise and using similar stimulation procedures (~13% *versus* ~6% in the present work) [9], suggesting that the present COPD patients exhibit a specific abdominal muscle response to exercise. In COPD, Hopkinson et al. [17] showed that >10% reduction in P_ga,tw_ occurred in only a fraction of patients (~35%) and the group mean reduction was ~7% after maximal incremental cycling. In the present study, about 25% of the patients had a >10% reduction in P_ga,tw_ and the mean reduction was ~6%, which is quite similar to the results of Hopkinson et al. [17]. Comparisons of Hopkinson data with the present results or previous studies regarding abdominal muscle fatigue are however difficult since thoracic stimulation was performed at TLC in Hopkinson et al. *versus* FRC in all other studies.

Our results showed that patients with small amplitude of dynamic hyperinflation during exercise exhibit larger reduction in P_ga,tw_ immediately after exhaustion (Figure 3C). These patients may have a greater abdominal muscle recruitment to limit the rise of EELV [43] and, consequently, may be more likely to develop abdominal muscle fatigue. Hopkinson et al. [17] reported similar dynamic hyperinflation in patients with significant and non-significant abdominal muscle fatigue. However in a recent study in patients with interstitial lung disease [44], more abdominal muscle fatigue was observed in patients who had greater fall in EELV during exercise. Although patients with interstitial lung disease did not exhibit significant increase in EELV during exercise, these findings might support the relationship between dynamic hyperinflation and abdominal muscle fatigue observed in the present study.

### Potential limitations

In our laboratory, both cervical and thoracic magnetic stimulation showed excellent within-day variability and have confirmed their ability to detect both inspiratory and expiratory muscle fatigue [22]. In the current work, we did not assess the supramaximality of magnetic stimulation to limit the number of stimulations. Supramaximal magnetic stimulation has been repeatedly confirmed for phrenic nerve and thoracic nerve root [18,22] and for femoral nerve [7,21] magnetic stimulation in both healthy subjects and COPD. To compare P_di,tw_ and P_ga,tw_ before and after exercise, it is also critical to perform stimulations at the same lung volume. Since P_oes_ before stimulations were similar at Baseline and after Exhaustion, we are confident that reductions in P_di,tw_ and P_ga,tw_ were not due to differences in lung volume. Another potential limitation of the present study is the absence of a healthy control group and thus, the fact that we had to rely on existing literature comparisons of the exercise response observed in COPD patients to normal responses obtained in healthy subjects.

### Generalization of the results and clinical implication

Our results emphasized the heterogeneous ventilatory abnormalities, neuromuscular function alterations and primary symptoms induced by exhaustive exercise in COPD patients [2–4]. Leg discomfort did not correlate with the magnitude of quadriceps contractile fatigue while such a relationship has been suggested in some [2,3] but not all [34] previous works on the topic. We also observed that both inspiratory and expiratory muscle fatigue did not correlate with dyspnea illustrating the multiple factors other than contractile fatigue (e.g. central factors) involved in the perception of leg discomfort and dyspnea [45]. Further studies with larger sample size including patients with various phenotypes (especially regarding pulmonary function, body composition, muscle weakness and comorbidities [46]) and various exercise response profiles (e.g. primary symptoms, amplitude of dynamic hyperinflation) are needed to understand the complex interplay of factors leading to exercise intolerance in COPD.

In conclusion we showed that patients with GOLD II-III COPD exhibit different amount of exercise-induced fatigue between respiratory muscle and locomotor muscles, with the largest amount of fatigue for the quadriceps, significant fatigue of the diaphragm and no significant change in abdominal muscle contractility at the group level. Respiratory muscle fatigue was shown to be more important in patients with smaller amount of dynamic hyperinflation. Hence, both respiratory and quadriceps fatigue should be considered to understand the physiopathology of exercise intolerance in COPD patients and to define efficient and individualized therapeutic intervention. 

## References

[1] Deschênes D, Pepin V, Saey D, LeBlanc P, Maltais F (2008) Locus of symptom limitation and exercise response to bronchodilation in chronic obstructive pulmonary disease. J Cardiopulm Rehabil Prev 28: 208-214. doi:10.1097/01.HCR.0000320074.73846.3b. PubMed: 18496322. 18496322

[2] Saey D, Michaud A, Couillard A, Côté CH, Mador MJ et al. (2005) Contractile fatigue, muscle morphometry, and blood lactate in chronic obstructive pulmonary disease. Am J Respir Crit Care Med 171: 1109-1115. doi:10.1164/rccm.200408-1005OC. PubMed: 15735055. 15735055

[3] Amann M, Regan MS, Kobitary M, Eldridge MW, Boutellier U et al. (2010) Impact of pulmonary system limitations on locomotor muscle fatigue in patients with COPD. Am J Physiol Regul Integr Comp Physiol 299: R314-R324. doi:10.1152/ajpregu.00183.2010. PubMed: 20445160. 20445160 PMC2904150

[4] Guenette JA, Webb KA, O'Donnell DE (2012) Does dynamic hyperinflation contribute to dyspnoea during exercise in patients with COPD? Eur Respir J 40: 322-329. doi:10.1183/09031936.00157711. PubMed: 22183485. 22183485

[5] O'Donnell DE, Webb KA (2008) The major limitation to exercise performance in COPD is dynamic hyperinflation. Journal of Appl Physiol 105: 753-757; discussion: 18678624. 18678624 10.1152/japplphysiol.90336.2008b

[6] van den Borst B, Slot IG, Hellwig VA, Vosse BA, Kelders MC et al. (2013) Loss of quadriceps muscle oxidative phenotype and decreased endurance in patients with mild-to-moderate COPD. Journal of Appl Physiol, 114: 1319–28. PubMed: 22815389. 22815389 10.1152/japplphysiol.00508.2012

[7] Mador MJ, Bozkanat E, Kufel TJ (2003) Quadriceps fatigue after cycle exercise in patients with COPD compared with healthy control subjects. Chest 123: 1104-1111. doi:10.1378/chest.123.4.1104. PubMed: 12684300. 12684300

[8] Butcher SJ, Lagerquist O, Marciniuk DD, Petersen SR, Collins DF et al. (2009) Relationship between ventilatory constraint and muscle fatigue during exercise in COPD. Eur Respir J 33: 763-770. doi:10.1183/09031936.00014708. PubMed: 19047319. 19047319

[9] Verges S, Schulz C, Perret C, Spengler CM (2006) Impaired abdominal muscle contractility after high-intensity exhaustive exercise assessed by magnetic stimulation. Muscle Nerve 34: 423-430. doi:10.1002/mus.20599. PubMed: 16810695. 16810695

[10] Johnson BD, Babcock MA, Suman OE, Dempsey JA (1993) Exercise-induced diaphragmatic fatigue in healthy humans. J Physiol 460: 385-405. PubMed: 8487201. 8487201 10.1113/jphysiol.1993.sp019477 PMC1175219

[11] Verges S, Sager Y, Erni C, Spengler CM (2007) Expiratory muscle fatigue impairs exercise performance. Eur J Appl Physiol 101: 225-232. doi:10.1007/s00421-007-0491-y. PubMed: 17546459. 17546459

[12] Mador MJ, Acevedo FA (1991) Effect of respiratory muscle fatigue on breathing pattern during incremental exercise. Am Rev Respir Dis 143: 462-468. doi:10.1164/ajrccm/143.3.462. PubMed: 2001052. 2001052

[13] Orozco-Levi M, Gea J, Lloreta JL, Félez M, Minguella J et al. (1999) Subcellular adaptation of the human diaphragm in chronic obstructive pulmonary disease. Eur Respir J 13: 371-378. doi:10.1183/09031936.99.13237199. PubMed: 10065684. 10065684

[14] Ottenheijm CA, Heunks LM, Hafmans T, van der Ven PF, Benoist C et al. (2006) Titin and diaphragm dysfunction in chronic obstructive pulmonary disease. Am J Respir Crit Care Med 173: 527-534. doi:10.1164/rccm.200507-1056OC. PubMed: 16339921. 16339921 PMC2662936

[15] Orozco-Levi M, Lloreta J, Minguella J, Serrano S, Broquetas JM et al. (2001) Injury of the human diaphragm associated with exertion and chronic obstructive pulmonary disease. Am J Respir Crit Care Med 164: 1734-1739. doi:10.1164/ajrccm.164.9.2011150. PubMed: 11719318. 11719318

[16] Polkey MI, Kyroussis D, Keilty SE, Hamnegard CH, Mills GH et al. (1995) Exhaustive treadmill exercise does not reduce twitch transdiaphragmatic pressure in patients with COPD. Am J Respir Crit Care Med 152: 959-964. doi:10.1164/ajrccm.152.3.7663810. PubMed: 7663810. 7663810

[17] Hopkinson NS, Dayer MJ, Moxham J, Polkey MI (2010) Abdominal muscle fatigue following exercise in chronic obstructive pulmonary disease. Respir Res 11: 15. doi:10.1186/1465-9921-11-15. PubMed: 20132549. 20132549 PMC2824704

[18] Mador MJ, Kufel TJ, Pineda LA, Sharma GK (2000) Diaphragmatic fatigue and high-intensity exercise in patients with chronic obstructive pulmonary disease. Am J Respir Crit Care Med 161: 118-123. doi:10.1164/ajrccm.161.1.9903010. PubMed: 10619807. 10619807

[19] Gosselink R, Troosters T, Decramer M (2000) Distribution of muscle weakness in patients with stable chronic obstructive pulmonary disease. J Cardiopulm Rehabil 20: 353-360. doi:10.1097/00008483-200011000-00004. PubMed: 11144041. 11144041

[20] Man WD, Hopkinson NS, Harraf F, Nikoletou D, Polkey MI et al. (2005) Abdominal muscle and quadriceps strength in chronic obstructive pulmonary disease. Thorax 60: 718-722. doi:10.1136/thx.2005.040709. PubMed: 15923239. 15923239 PMC1747513

[21] Verges S, Maffiuletti NA, Kerherve H, Decorte N, Wuyam B et al. (2009) Comparison of electrical and magnetic stimulations to assess quadriceps muscle function. J Appl Physiol (1985) 106: 701-710. PubMed: 18756009. 18756009 10.1152/japplphysiol.01051.2007

[22] Verges S, Bachasson D, Wuyam B (2010) Effect of acute hypoxia on respiratory muscle fatigue in healthy humans. Respir Res 11: 109. doi:10.1186/1465-9921-11-109. PubMed: 20701769. 20701769 PMC2929221

[23] (2013) Global Initiative for Chronic Obstructive Lung Disease (GOLD). Global Strategy for the Diagnosis, Management and Prevention of COPD. Available http://www.goldcopd.org/.

[24] Miller MR, Hankinson J, Brusasco V, Burgos F, Casaburi R et al. (2005) Standardisation of spirometry. Eur Respir J 26: 319-338. doi:10.1183/09031936.05.00034805. PubMed: 16055882. 16055882

[25] Bachasson D, Millet GY, Decorte N, Wuyam B, Levy P et al. (2013) Quadriceps function assessment using an incremental test and magnetic neurostimulation: A reliability study. J Electromyogr Kinesiol, 23: 649–58. PubMed: 23265662. 23265662 10.1016/j.jelekin.2012.11.011

[26] Kufel TJ, Pineda LA, Mador MJ (2002) Comparison of potentiated and unpotentiated twitches as an index of muscle fatigue. Muscle Nerve 25: 438-444. doi:10.1002/mus.10047. PubMed: 11870723. 11870723

[27] Similowski T, Fleury B, Launois S, Cathala HP, Bouche P et al. (1989) Cervical magnetic stimulation: a new painless method for bilateral phrenic nerve stimulation in conscious humans. J Appl Physiol (1985) 67: 1311-1318. PubMed: 2676953. 2676953 10.1152/jappl.1989.67.4.1311

[28] Mador MJ, Magalang UJ, Kufel TJ (1994) Twitch potentiation following voluntary diaphragmatic contraction. Am J Respir Crit Care Med 149: 739-743. doi:10.1164/ajrccm.149.3.8118645. PubMed: 8118645. 8118645

[29] Laghi F, Topeli A, Tobin MJ (1998) Does resistive loading decrease diaphragmatic contractility before task failure? J Appl Physiol (1985) 85: 1103-1112. PubMed: 9729589. 9729589 10.1152/jappl.1998.85.3.1103

[30] Hamnegard CH, Wragg S, Mills G, Kyroussis D, Road J, et al. (1995) The effect of lung volume on transdiaphragmatic pressure. Eur Respir J 8: 1532-1536 8575580

[31] Killian KJ, Leblanc P, Martin DH, Summers E, Jones NL et al. (1992) Exercise capacity and ventilatory, circulatory, and symptom limitation in patients with chronic airflow limitation. Am Rev Respir Dis 146: 935-940. doi:10.1164/ajrccm/146.4.935. PubMed: 1416421. 1416421

[32] Decorte N, Lafaix PA, Millet GY, Wuyam B, Verges S (2012) Central and peripheral fatigue kinetics during exhaustive constant-load cycling. Scand J Med Sci Sports 22: 381-391. doi:10.1111/j.1600-0838.2010.01167.x. PubMed: 20807390. 20807390

[33] Amann M, Dempsey JA (2008) Locomotor muscle fatigue modifies central motor drive in healthy humans and imposes a limitation to exercise performance. J Physiol 586: 161-173. PubMed: 17962334. 17962334 10.1113/jphysiol.2007.141838 PMC2375542

[34] Saey D, Debigare R, LeBlanc P, Mador MJ, Cote CH et al. (2003) Contractile leg fatigue after cycle exercise: a factor limiting exercise in patients with chronic obstructive pulmonary disease. Am J Respir Crit Care Med 168: 425-430. doi:10.1164/rccm.200208-856OC. PubMed: 12714348. 12714348

[35] Mador MJ, Magalang UJ, Rodis A, Kufel TJ (1993) Diaphragmatic fatigue after exercise in healthy human subjects. Am Rev Respir Dis 148: 1571-1575. doi:10.1164/ajrccm/148.6_Pt_1.1571. PubMed: 8256903. 8256903

[36] Verges S, Lenherr O, Haner AC, Schulz C, Spengler CM (2007) Increased fatigue resistance of respiratory muscles during exercise after respiratory muscle endurance training. Am J Physiol Regul Integr Comp Physiol 292: R1246-R1253. PubMed: 17068160. 17068160 10.1152/ajpregu.00409.2006

[37] Walker DJ, Walterspacher S, Schlager D, Ertl T, Roecker K et al. (2011) Characteristics of diaphragmatic fatigue during exhaustive exercise until task failure. Respir Physiol Neurobiol 176: 14-20. doi:10.1016/j.resp.2011.01.009. PubMed: 21281744. 21281744

[38] Froyd C, Millet GY, Noakes TD (2013) The development of peripheral fatigue and short-term recovery during self-paced high-intensity exercise. J Physiol 591: 1339-1346. doi:10.1113/jphysiol.2012.245316. PubMed: 23230235. 23230235 PMC3607875

[39] De Troyer A (1997) Effect of hyperinflation on the diaphragm. Eur Respir J 10: 708-713. PubMed: 9073010. 9073010

[40] McKenzie DK, Butler JE, Gandevia SC (2009) Respiratory muscle function and activation in chronic obstructive pulmonary disease. J Appl Physiol (1985) 107: 621-629. doi:10.1152/japplphysiol.00163.2009. PubMed: 19390004. 19390004

[41] Fitch S, McComas A (1985) Influence of human muscle length on fatigue. J Physiol 362: 205-213. PubMed: 4020687. 4020687 10.1113/jphysiol.1985.sp015671 PMC1192890

[42] Caron MA, Debigaré R, Dekhuijzen PN, Maltais F (2009) Comparative assessment of the quadriceps and the diaphragm in patients with COPD. Journal of Appl Physiol 107: 952-961. doi:10.1152/japplphysiol.00194.2009. PubMed: 19359618. 19359618

[43] Abraham KA, Feingold H, Fuller DD, Jenkins M, Mateika JH et al. (2002) Respiratory-related activation of human abdominal muscles during exercise. J Physiol 541: 653-663. doi:10.1113/jphysiol.2001.013462. PubMed: 12042369. 12042369 PMC2290343

[44] Elia D, Kelly JL, Martolini D, Renzoni EA, Boutou AK et al. (2013) Respiratory Muscle Fatigue following Exercise in Patients with Interstitial Lung Disease. Respiration, 85: 220–7. PubMed: 22813885. 22813885 10.1159/000338787

[45] O'Donnell DE, Ora J, Webb KA, Laveneziana P, Jensen D (2009) Mechanisms of activity-related dyspnea in pulmonary diseases. Respir Physiol Neurobiol 167: 116-132. doi:10.1016/j.resp.2009.01.010. PubMed: 19450767. 19450767

[46] Han MK, Agusti A, Calverley PM, Celli BR, Criner G et al. (2010) Chronic obstructive pulmonary disease phenotypes: the future of COPD. Am J Respir Crit Care Med 182: 598-604. doi:10.1164/rccm.200912-1843CC. PubMed: 20522794. 20522794 PMC6850732

